# Optimizing Wnt activation in fetal calf serum (FCS)-free organoid expansion media

**DOI:** 10.3389/ftox.2025.1504469

**Published:** 2025-05-14

**Authors:** Esther J. M. Liefting, Jeffrey J. Bajramovic

**Affiliations:** 3Rs Centre Utrecht, Utrecht University, Utrecht, Netherlands

**Keywords:** FCS-free medium, expansion medium, Wnt signaling, organoids, regenerative medicine, the 3Rs

## Abstract

Organoid technology can revolutionize biomedical research by increasing the translational value of experimental results while at the same time reducing the need for experimental animal use. However, in most cases the organoid culture workflow relies on expansion media that contain fetal calf serum (FCS). The production of FCS causes animal suffering, and the use of it is hampered by factors that negatively impact the reproducibility (such as the large inter-batch variation and the undefined composition of FCS), relevance (such as the induction of a non-physiological cellular phenotype), as well as the clinical translatability (such as the potential to cause xeno-immunization or to contain xenogeneic pathogens). There is thus a strong impetus to find animal-free alternatives to the use of FCS. Most contemporary expansion media for organoid culture are not FCS-free. This is mainly contributable to the use of FCS for the recombinant production of the growth factor Wnt3A. Wnt3A-conditioned medium is added to expansion media to induce Wnt signaling, which is necessary for organoid proliferation. In turn, FCS is pivotal to stabilize and solubilize the Wnt3A protein, and not perse for the survival, adhesion or proliferation of cells. This mini-review explores alternative methods to induce Wnt signaling in organoid expansion media, encompassing the use of soluble Wnt mimetics, the use of carriers, and the use of small molecule inhibitors. Ultimately, alternative Wnt activation approaches for different experimental goals are reviewed and discussed.

## Introduction

Organoids are three-dimensional cellular models that can be used for fundamental research and clinical regenerative medicine purposes. By using organoids as an experimental model, the need for animal models can be reduced (Kim et al., 2020). In addition, clinical applications of organoids can reduce the need for donor organs, for example in metabolic liver diseases (Schneemann et al., 2020; Tsuchida et al., 2020).To reproducibly expand organoids for both experimental as well as clinical purposes, defined cell culture media are necessary. For most organoid culture media, Dulbecco’s Modified Eagle’s Medium (DMEM) is used as a basis. DMEM contains amino acids, vitamins, glucose, sodium pyruvate and a buffer system to maintain a physiological pH. The growth factor Wnt3A, most often added in the form of Wnt3A-conditioned medium, is a necessary supplement for expansion media to induce proliferation and organoid outgrowth. Wnt3A-conditioned medium is obtained by culturing a cell line that recombinantly expresses Wnt3A. To facilitate release and stabilization of recombinantly produced Wnt3A, fetal calf serum (FCS) is added to the cell culture medium (Pleguezuelos‐Manzano et al., 2020; VanDussen et al., 2019; Willert et al., 2003).

The use of FCS in cell culture protocols however goes accompanied by experimental, clinical and ethical concerns. The production of FCS is characterized by large inter-batch variations, and the undefined composition of the complex biological fluid negatively impacts the reproducibility of experimental results. Furthermore, exposure of cells in culture to FCS induces a proliferative phenotype which may be non-physiological (Timmerman et al., 2022). In addition, FCS has been suggested to induce cellular senescence in pancreatic organoids, thereby inhibiting long term culture (Mazzella et al., 2023; Seino et al., 2018; Rauch et al., 2011). Growth obstruction is especially detrimental for protocols that require organoid outgrowth from single cells (e.g. CRISPR-Cas9-mediated genome engineering) (Drost et al., 2015).

The use of FCS also poses risks for the clinical translatability (Schneemann et al., 2020; Tsuchida et al., 2020). Regulatory authorities promote switching to FCS-free media for organoids grown for clinical purposes, because of the potential presence of infectious agents and of the risk of inducing xeno-immunization in patients (Chimenti et al., 2014; Mackensen et al., 2000; Selvaggi et al., 1997; Bauman et al., 2018). Untreated FCS therefore does not comply with European Good Manufacturing Practices (GMP), and even gamma-irradiation does not eliminate all clinical risks (Chimenti et al., 2014; Giannitti et al., 2022). Xeno-immunization against FCS proteins has been demonstrated in clinical trials, causing anaphylaxis and fever reactions in patients (Mackensen et al., 2000; Selvaggi et al., 1997). Therefore xeno-free chemically defined media are encouraged in research fields focusing on developing clinical cellular therapies, like the multipotent mesenchymal stromal cells (MSCs) and the human embryonic stem cells (hESC) fields, and more basal organoid research will likely follow suit (Lee et al., 2022; Palamà et al., 2023).

Finally, the production of FCS also raises ethical concerns (Barosova et al., 2021). FCS is collected via cardiac puncture at a stage when the calf fetus can already feel pain and distress (Bauman et al., 2018). Approximately 3 calves are needed to obtain 1 liter of FCS, which results in the killing of around 2 million fetal calves yearly (Bauman et al., 2018).

The reasons to stop using FCS are thus numerous, but replacing FCS to produce Wnt3A-conditioned medium remains challenging as FCS is necessary for the solubilization of Wnt3A protein, and greatly enhances protein stability. Although FCS-free Wnt activation approaches have been studied for organoid expansion, a comparative review on the different strategies has thus far been lacking. This review provides such an overview, taking different experimental goals into account.

**FIGURE 1:**
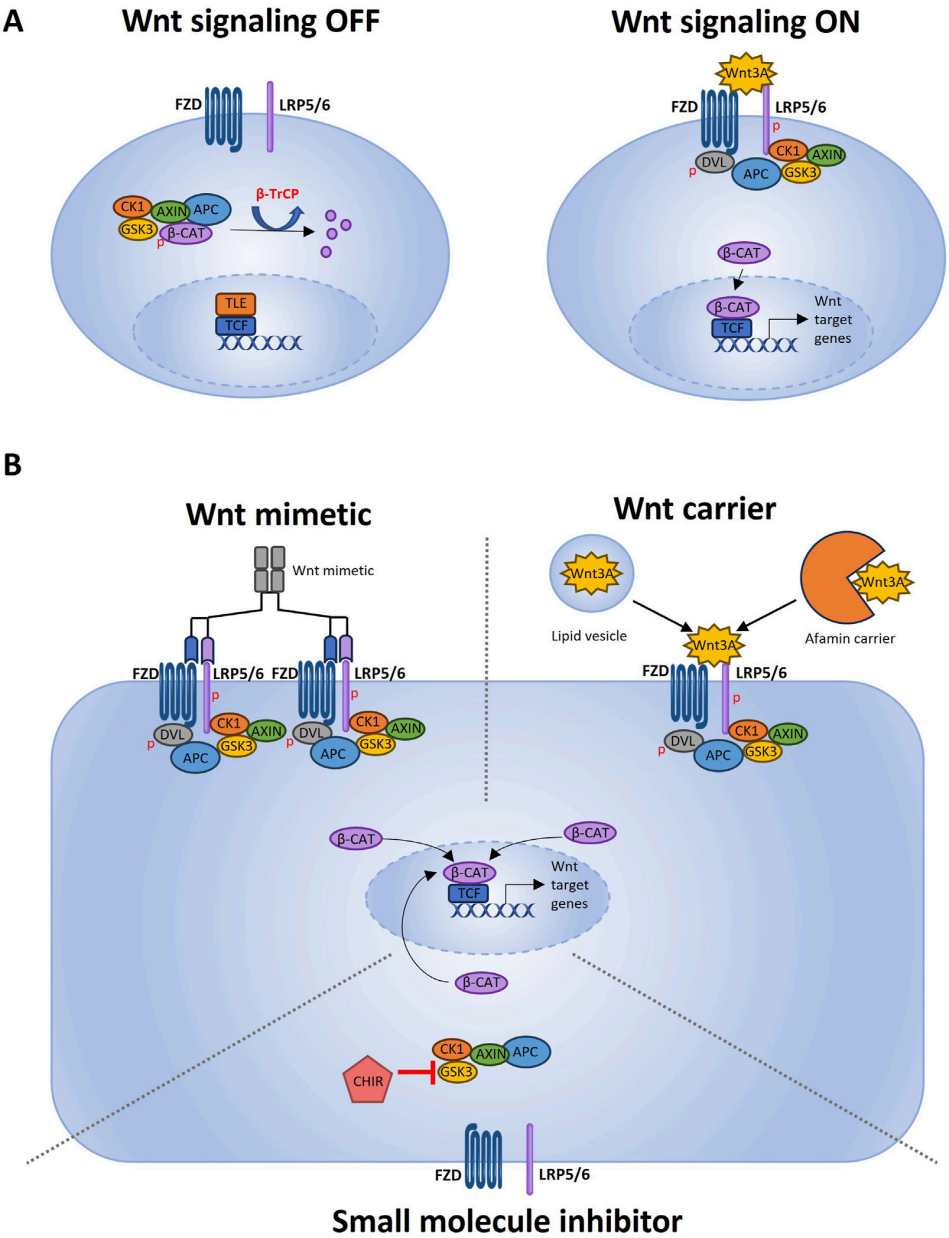
Wnt pathway activation and different FCS-free strategies to induce Wnt activation. **(A)** Schematic representation of the Wnt signal transduction cascade adapted from Patel et al. (2019) using Microsoft PowerPoint. When Wnt signaling is OFF, the transcription factor β-catenin is actively degraded. The β-catenin destruction complex consisting of GSK3, CK1, APC and Axin creates a β-TrCP recognition site on β-catenin by phosphorylation (Stamos and Weis, 2013). β-TrCP is a ubiquitin ligase which marks β-catenin for proteasomal degradation. Wnt signaling is ON when a ligand such as Wnt3A binds to the FZD transmembrane receptor and the LRP5/6 co-receptor. Upon activation of FZD and LRP5/6, the downstream regulator Dishevelled (Dvl) forms a complex with FZD (Romero et al., 2024). This binding of Dvl triggers the recruitment of the scaffolding protein Axin and GSK3, resulting in a clustered signalosome (Zeng et al., 2008). Phosphorylation of the LPR5/6 co-receptor by CK1 cause more Axin and GSK3 binding (Niehrs and Shen, 2010). Since Axin and GSK3 are part of the β-catenin destruction complex, less cytosolic Axin and GSK3 will lead to more stable cytosolic β-catenin. When stable β-catenin accumulates in the cytoplasm, it will migrate to the nucleus where it functions as a transcription factor. By removing the transcriptional co-repressors groucho/transducin like enhancer of split (TLE) from the transcription start site, β-catenin is able to bind to the DNA and, together with DNA-binding factors of the TCF/LEF family, can induce gene expression (Hrckulak et al., 2016; Ramakrishnan et al., 2018).**(B)** This original Figure made using Microsoft PowerPoint depicts different strategies for inducing Wnt signaling in FCS-free expansion media (Kim et al., 2020). Wnt mimetics that can be multivalent and bind several receptors to potently induce Wnt activation (Schneemann et al., 2020). Wnt3A carriers that can solubilize and stabilize recombinant Wnt3A, which can bind to the FZD and LRP5/6 receptors to activate Wnt signaling (Tsuchida et al., 2020). CHIR that can activate Wnt signaling by inhibiting GSK3. GSK3 is part of the β-catenin destruction complex, so GSK3 inhibition causes less β-catenin destruction and, as a result, more active Wnt signaling.

Wnt3A is such a popular organoid medium supplement because it can bind to all FZD receptors to induce canonical Wnt signaling, as opposed to other -more selective- Wnt ligands (Voloshanenko et al., 2017). Active canonical Wnt signaling is characterized by inhibition of the β-catenin destruction complex. This results in more stable cytosolic β-catenin translocating to the nucleus (Niehrs, 2012). Figure 1 illustrates the Wnt3A-induced activation of the canonical Wnt pathway (Figure 1A), as well as the further below-described FCS-free strategies to achieve a similar effect (Figure 1B).

There are several possibilities to obtain serum-free Wnt3A. The most straightforward one is to purify recombinant Wnt3A protein by using Cu2+-charged Sepharose beads (Witkowski et al., 2015). This is however expensive, difficult to scale up, and requires maintaining a recombinant cell line culture (Witkowski et al., 2015; Wilson et al., 2020). Alternatively, commercial purified recombinant Wnt3A can be used. As per product information available via the manufacturers’ website or via the product spec sheet, some manufacturers grow these cell lines using FCS, while others disclose using serum-free defined media, resulting in GMP-grade proteins that are suitable for clinical applications as well.

However, the addition of recombinant, serum-free, Wnt3A to expansion medium is not a viable option because of problems with protein solubility and stability. The hydrophobic nature of Wnt3A protein causes aggregation in aqueous solutions in the absence of serum or detergent (Willert et al., 2003; Willert, 2008). In addition, macro biomolecules that are present in FCS, like heparan sulfate proteoglycans (HPSGs), stabilize and preserve Wnt3A activity (Fuerer et al., 2010). Supplementation of Wnt3A to FCS-free DMEM medium resulted in an instant decrease of Wnt3A signaling by 3-fold, and further incubation resulted in a complete loss of signaling (Fuerer et al., 2010). By contrast, Wnt3A signaling activity was only marginally reduced after 6 h of incubation in medium supplemented with 10% FCS, due to HPSGs stabilization (Fuerer et al., 2010).

### Wnt mimetics: soluble Wnt agonists

To circumvent the hydrophobic nature of full-length Wnt proteins, recombinant proteins that mimic Wnt activation by binding to the FZD and LRP5/6 receptor can be used (Figure 1B). These Wnt mimetics are structurally unrelated to natural Wnt and can either be antibody-based or binding domain-based. Multiple studies have explored antibody-based Wnt mimetics, also called Wnt surrogates, and experimented with different approaches. The first generation of Wnt surrogates consisted of an Fc-antibody fragment for FZD receptor binding, and the C-terminal domain of Wnt antagonist DKK1 for LRP5/6 binding. This was demonstrated to induce similar or superior growth of organoids derived from the pancreas, colon and stomach (corpus) as compared to Wnt3A-conditioned medium control (Janda et al., 2017). Wnt surrogates were less active in supporting the growth of liver organoids when compared to the Wnt3A-conditioned medium control (Janda et al., 2017). Later studies on multivalent Wnt surrogates demonstrated that simultaneous binding and recruitment of multiple FZD receptors resulted in even more potent pathway activation (Chen et al., 2020; Tao et al., 2019). To increase the stability of Wnt surrogates, a full length IgG antibody can be used (Post et al., 2023). This has been demonstrated to be more stable than the wildtype protein, and had a similar PK/PD profile to antibody molecules in *in vivo* studies. Potential immunogenicity of Wnt surrogates was, however, not investigated (Post et al., 2023). Antibody-derived Wnt mimetics have been demonstrated to support organoid proliferation and growth for human kidney tubular epithelial organoids and pancreatic, colon, intestine, stomach, esophagus, ovarian, breast and liver organoids (Janda et al., 2017; Chen et al., 2020; Tao et al., 2019; Post et al., 2023).

Binding domain-based Wnt mimetics have also been demonstrated to be similarly or more effective in supporting organoid outgrowth than Wnt3A-conditioned medium (Miao et al., 2020). These Wnt mimetics contain designed repeat protein binders for high affinity FZD receptor binding (Miao et al., 2020). This effectively supported organoid proliferation for human kidney tubular epithelial organoids and pancreas, colon, stomach, ovarian, breast and liver organoids (Miao et al., 2020).

An important advantage of Wnt mimetics is that they have been reported to be able to support efficient organoid outgrowth from single cells for pancreas, stomach, and colon organoids (Post et al., 2023; Miao et al., 2020). When using Wnt3A-conditioned medium, success rates for single cell outgrowth are low. Using Wnt mimetics for improved single cell organoid outgrowth can therefore especially benefit protocols for high-throughput experiments with organoids, such as CRISPR-Cas9-mediated genome editing (Drost et al., 2015) and the establishment of organoid biobanks (van de Wetering et al., 2015). Wnt mimetics thus provide a modular platform that allows the user to design a recombinant protein to specifically induce Wnt signaling (Chen et al., 2020; Tao et al., 2019; Post et al., 2023). Natural Wnt molecules are cross-reactive for the different subtypes of FZD receptors, making it challenging to investigate pathway mechanisms. Using recombinant proteins based on antibodies or on binding domains, specific subtypes of FZD receptors can be activated which allows further scrutinization of the Wnt pathway, which can be considered as another advantage of Wnt mimetics. However, the production process of Wnt mimetics is not yet entirely FCS-free. For both antibody- as well as binding domain-based mimetics, the supportive cell lines are grown on medium with 10% FCS (Post et al., 2023; Miao et al., 2020).

### Wnt3A carriers

Another strategy to induce Wnt3A-mediated signaling is by using a molecular carrier that binds the Wnt3A protein to enhance its solubility and stability (Figure 1B). Such a carrier has to specifically protect the palmitoylated Cys77 and the palmitoylated Ser209 moieties of the Wnt3A molecule. Palmitoylated Cys77 is essential to induce internalization of the LRP6 co-receptor (Haack et al., 2020; Komekado et al., 2007), and palmitolated Ser209 is required for Wnt3A secretion (Takada et al., 2006).

Lipids are good candidate carriers for this purpose. They have been widely used for the delivery of small molecules and DNA, and are well tolerated by cells (Dhamdhere et al., 2014). Wnt3A-carrying lipids can effectively stabilize Wnt3A in culture (Tüysüz et al., 2017), and were able to retain 65% of the Wnt3A activity, after a 6h incubation at 37°C. By comparison, when pure Wnt3A (solubilized by using a detergent) was used, all activity was lost after 6 h (Dhamdhere et al., 2014). In addition, lipid carriers supported higher proliferation rates of human duodenum organoids as compared to purified Wnt3A control (Tüysüz et al., 2017). On top of this, the affinity of Wnt3A proteins for lipid carriers can also be exploited for purifying Wnt3A (Tüysüz et al., 2017). More than 80% of Wnt3A proteins were recovered from a low purity Wnt3A suspension by isolating the liposomes (Tüysüz et al., 2017). This process removes almost all contaminants, except for Bovine Serum Albumin (BSA), which means that the medium is not xeno-free (Tüysüz et al., 2017). Further research is necessary to reduce the use of BSA in future studies.

Another candidate carrier for Wnt3A is the glycoprotein afamin, which is found in blood serum. Afamin can form 1:1 complexes with most of the 19 known Wnt proteins to maintain their biological activity (Mihara et al., 2016; Naschberger et al., 2017). The crystal structure of human afamin reveals a binding pocket for the palmitoylated Ser209 of Wnt3A (Naschberger et al., 2017). By adding purified recombinant afamin to the medium of Wnt3A-expressing HEK cells, FCS-free Wnt3A secretion was facilitated (Naschberger et al., 2017). FCS-free, afamin-stabilized, Wnt3A medium supported the maintenance of pancreatic organoids for over 8 months, whereas supplements that contained FCS induced senescence in the stem cells (Seino et al., 2018). Without afamin as a carrier, and with a solubilizing detergent, Wnt3A was stable for 6 h (Mihara et al., 2016). By contrast, when similar concentrations of afamin-Wnt3A complex were used, this supported organoid growth until the next passage, 7 days later (Mihara et al., 2016). The afamin carrier stabilized and preserved Wnt3A activity even after several weeks of storage (Mihara et al., 2016).

Although these reports are positive on the effects of afamin as a carrier, it has also been described that afamin was unable to conserve the activity of purified Wnt3A (Tüysüz et al., 2017). This discrepancy may be explained by technical issues. In the original study, a co-expression method was used to investigate the afamin-Wnt3A-complex (Mihara et al., 2016), whereas in the study of Tüysüz et al. only afamin was expressed recombinantly (Tüysüz et al., 2017). It has therefore been hypothesized that either the recombinant afamin from Tüysüz *et al.* lacked essential modifications, or that Wnt3A stabilization can only occur when afamin and Wnt3A directly interact with each other during their biosynthesis (Tüysüz et al., 2017). More detailed biosynthesis studies are needed to shed light on this.

### Small molecule inhibitors

Finally, Wnt signaling can also be induced by using the small molecule inhibitor Chiron99021 (CHIR) (Figure 1B). CHIR inhibits GSK3, which is part of the beta-catenin destruction complex. By inhibiting GSK3, less beta-catenin is broken down and as a consequence Wnt signaling will be induced. CHIR is more stable, cheaper and easier to use, than Wnt ligands (Bain et al., 2007). CHIR successfully activated Wnt signaling in a chemically defined, open access MCDB131-based, medium for the expansion and long term culture of pancreatic precursors (Konagaya and Iwata, 2019). In a different study, a cocktail of small molecules like CHIR, blebbistatin and forskolin was used to generate liver organoids (Wang et al., 2020). This medium with small molecules is economical, convenient and sufficient to activate essential pathways for bipotential liver organoid culture (Wang et al., 2020). However, CHIR cannot always 1:1 replace Wnt ligands. In a comparative study, human duodenum organoids could not be expanded as effectively by using CHIR as by using purified Wnt3A proteins (Tüysüz et al., 2017). In addition, the use of CHIR can cause undesired downstream effects, since GSK3 also regulates many other pathways (Jope and Johnson, 2004). CHIR can thus certainly be considered as a defined and FCS-free way to activate Wnt signaling, but it will also affect the regulation of other cellular pathways. Depending on your specific organoid application this may, or may not, be a problem, and side-by-side testing data are necessary to make further distinctions.

## Discussion

To expand organoids, Wnt3A is an essential growth factor which is often produced in media that contain FCS. FCS helps to solubilize and stabilize Wnt3A, but the use of FCS goes accompanied by ethical and scientific concerns. It has however proven difficult to replace the use of FCS in the production process of Wnt3A, since supportive cell lines need FCS for survival and for the release of Wnt3A. In this review we explored the use of Wnt mimetics, Wnt carriers and small molecules as animal-free replacements for FCS, taking into account effectiveness, specificity, cost and the type(s) of organoids used for testing (Table 1).

**TABLE 1:** Comparison of FCS-free, Wnt activation strategies in organoid expansion media.

Method	Organoid outgrowth efficiency	Completely serum-free production	Specific targeting	Organoid types	Associated costs*
Wnt3A mimetics	High	Possible	Yes	Pancreas, colon, intestine, esophagus, stomach, hepatocyte, salivary gland, ovarian and breast	High
Wnt3A + lipid carrier	Medium	Possible	Yes	Duodenum, jejunum	High - Intermediate
CHIR	Low	Yes	No	Pancreatic precursor, liver, cerebral	Low
Wnt3A-conditioned medium supplement	Medium	No	Yes	Pancreas, intestine	Low

*Comparing the costs of the different methods is difficult, since not all methods use the same basal medium and not all methods have been described to work for all organoid types. In addition, the recommended concentrations of the supplementation methods differ for different organoid types. We have therefore chosen to base our cost estimations on the Wnt-activating supplementation necessary for 10 mL of expansion media to support pancreatic or duodenal organoids, since these organoid types were reported on most frequently.

The cost of using commercial Wnt mimetics (at a described effective dose of 200 µM) (Willert, 2008), is at the time of publishing around €1750 (Thermofisher). The cost of in-house production of Wnt mimetics cannot be determined, since the production yields of recombinant cell lines and purification methods used are not known and may vary between labs (Chen et al., 2020).

The total cost of using lipid carrier-stabilized Wnt is around €1,400. Purified recombinant Wnt3A (at a dose of 11 µg/mL) costs €1,100 (R&D systems). Lipid carrier mix (used at a dose of 15 mM) costs around €300 (from e.g. Sigma, Echelon) (Takada et al., 2006). If recombinant Wnt3A is produced in-house the costs will be much lower. This will also allow for testing the FCS-free Wnt3A production in the presence of afamin. Initial investments to establish in-house production are associated with the purchase of a recombinant cell line expressing Wnt3A and with the investment in time to set up the system. Further downstream costs of in-house production of recombinant Wnt3A cannot be exactly determined, since the production yields of recombinant cell lines and purification methods may vary between labs.

The cost of using CHIR-99021 (at a described effective dose of 3 μM, StemCell Research) is around €150 at the time of publishing (Bain et al., 2007).

For reference purposes: the cost of Wnt3A-conditioned medium (50/50 vol/vol) is €36 (Merck) (Pleguezuelos‐Manzano et al., 2020).

Wnt mimetics can support organoid outgrowth with similar or better efficacy than conventional Wnt3A-conditioned medium. The Wnt mimetic platform allows for effective and precise activation of Wnt pathways, because multivalency and simultaneous binding of multiple receptors can result in more potent pathway activation than is achieved by natural Wnt3A (Chen et al., 2020; Tao et al., 2019; Post et al., 2023). The potency of Wnt mimetics to support organoid outgrowth has been confirmed for many different organoid types (Janda et al., 2017; Chen et al., 2020; Post et al., 2023; Miao et al., 2020), although differential activity for some organoid types was observed (Janda et al., 2017). This may be due to differential requirements for FZD triggering between different organoids or to unknown factors that may be present in the Wnt3A-conditioned medium (Janda et al., 2017). The broad application combined with the potency to support organoid outgrowth for singe cells make Wnt mimetics a good choice for high-throughput organoid workflows, such as CRISPR-Cas9-mediated genome editing (Drost et al., 2015) and for the establishment of organoid biobanks (van de Wetering et al., 2015). However, out of the three FCS replacement approaches described here, Wnt mimetics are the most expensive (Table 1). When acquired commercially they are expensive, and also when produced in-house the initial investments in time and money are high because of the need to establish and maintain a recombinant cell line combined with the use of expensive antibody purification resins (Post et al., 2023).

The use of carriers to solubilize and stabilize Wnt3A proteins can be considered as a more economical option, especially in combination with in-house produced recombinant Wnt3A (Table 1). Although there are initial investments in money and time associated with the purchase or with the establishment of a recombinant cell line and protein purification columns, further maintenance is relatively cheap. An additional advantage is that the use of carrier-stabilized recombinant Wnt3A closely mimics the *in vivo* situation Whereas the use of lipids as carriers for Wnt3A has been described to enable activation of canonical Wnt signaling and maintenance of duodenum organoids (Tüysüz et al., 2017), reports on the use of afamin are equivocal. Technical issues may underlie these differences, and more research should be performed to be able to conclusively decide on the suitability of either carrier for specific applications (Tüysüz et al., 2017; Mihara et al., 2016).

The small molecule inhibitor CHIR represents the least precise way to induce Wnt signaling in organoids. On the other hand, it is by far the least expensive alternative to the use of Wnt3A-conditioned medium (Table 1) since it does not require the purchase or maintenance of recombinant cell lines and associated purification steps. CHIR inhibits GSK3, which is also involved in signaling by a variety of other pathways. The use of CHIR has been proven effective for certain types of organoids only (Tüysüz et al., 2017; Konagaya and Iwata, 2019; Wang et al., 2020), and can be considered for liver and pancreatic organoids in particular. The molecular mechanism(s) behind this selectivity are currently unknown and whether this is e.g. correlated to a differential dependency of liver and pancreatic organoids on events downstream of GSK3 signaling as compared to other organoid types remains to be established. Even when CHIR is used for the expansion of liver and pancreatic organoids specific attention should be paid to potential effects on other GSK3-dependent signaling pathways (Jope and Johnson, 2004).

Studies that have directly compared the suitability of different Wnt activation methods for the expansion of different types of organoids are currently lacking, rendering conclusive statements impossible. Such studies could assess the potential to support organoid outgrowth from single cells, the efficacy in % organoid outgrowth as well as expansion speed related to Wnt3A-conditioned medium as a reference. Preferably, to ensure replicability results should be compared to the same batch of Wnt3A-conditoned medium. By summarizing the data available, we hope to stimulate the discussion and the development of efficient testing strategies for the replacement of FCS from organoid expansion media.

Although it will require an initial investment to find the best FCS replacement strategy for specific purposes, there are important benefits to be gained. The use of a defined FCS-free expansion medium will allow optimization of organoid yield and interlaboratory replicability. In addition, when gene-edited organoids are to be transplanted back into the patient, for example to ameliorate metabolic liver diseases, it is important that expansion and culture media are GMP-compliant and xeno-free to avoid complications (Schneemann et al., 2020; Tsuchida et al., 2020; Mackensen et al., 2000; Selvaggi et al., 1997; Giannitti et al., 2022; Macy et al., 1989). As mentioned before, complications like anaphylaxis and fever reactions after cell therapy treatment have been reported due to xeno-immune responses directed against FCS-derived proteins (Mackensen et al., 2000; Selvaggi et al., 1997). Currently three clinical trials have been registered that involve organoids for transplantation or cell based therapy. These studies investigate the regenerative potential of intestinal organoids for ulcerative colitis treatment (clinical trial UMIN000030117, Japanese Registry), islet organoids for diabetes treatment (clinical trial NCT06415643) and salivary gland organoids for the treatment of radiation-induced xerostomia (clinical trial NCT04593589). Although it is not disclosed which organoid expansion medium were used in these studies, the scientists of the ulcerative colitis study specifically mention the development of clinical-grade culture methods as one of their challenges for safe in-human studies (Takebe et al., 2018; Shimizu et al., 2019). Finally, more efficient organoid outgrowth will also help with culturing gene-edited organoids from single cells, which has a very low success rate when the conventional Wnt3A-conditioned medium is used. Optimization of the culture of gene-edited organoids in particular has great potential to reduce the need for animal experiments and animal-derived products in e.g. drug testing, drug discovery, personalized medicine and disease modeling.
